# Functional alignment in robotic‐assisted total knee arthroplasty for valgus deformity achieves safe coronal alignment and excellent short‐term outcomes

**DOI:** 10.1002/ksa.12585

**Published:** 2025-01-17

**Authors:** Pietro Gregori, Christos Koutserimpas, Vasileios Giovanoulis, Cécile Batailler, Elvire Servien, Sébastien Lustig

**Affiliations:** ^1^ Orthopaedics Surgery and Sports Medicine Department, FIFA Medical Center of Excellence, Croix Rousse Hospital, Hospices Civils de Lyon Lyon North University Hospital Lyon France; ^2^ Fondazione Policlinico Universitario Campus Bio‐Medico Roma Italy; ^3^ Univ Lyon, Claude Bernard Lyon 1 University, IFSTTAR LBMC UMR_T9406 Lyon France; ^4^ LIBM‐EA 7424, Interuniversity Laboratory of Biology of Mobility Claude Bernard Lyon 1 University Lyon France

**Keywords:** functional alignment, knee phenotype, robotic knee replacement, valgus knee

## Abstract

**Purpose:**

Functional alignment (FA) in total knee arthroplasty (TKA) prioritizes soft tissue balancing and anatomical restoration without systematic correction to neutral alignment. Most studies have focused on varus deformity, with little evidence available about FA in valgus deformity. The hypothesis of the present study was that FA in robotic‐assisted TKA for valgus deformity would demonstrate correction of the coronal alignment and yield satisfactory short‐term outcomes.

**Methods:**

This retrospective study included 58 patients with valgus coronal alignment (hip–knee–angle [HKA] ≥ 183°) who underwent robotic‐assisted TKA using the FA technique with a minimum of 1‐year follow‐up. Outcomes were assessed through the Knee Society Score (KSS), Oxford Knee Score (OKS), Forgotten Joint Score (FJS) and radiographic measurements of alignment and phenotypes. Complication and revision rates were also analyzed.

**Results:**

The cohort included 39 females and 19 males with a median age of 70. Post‐operatively, 86.2% of cases achieved coronal alignment within the safe zone (HKA 177–183°). Significant improvements were observed in KSS (part 1: 69.5–95, part 2: 65–94, *p* < 0.001), while OKS and FJS exhibited optimal outcomes. Two complications were recorded: one aseptic loosening (1.7%) and one early infection (1.7%). Kaplan–Meier survival analysis indicated favourable implant survivorship at a median follow‐up of 18 months.

**Conclusion:**

FA in image‐based robotic TKA is a safe and effective approach for patients with valgus deformity. This procedure resulted in a modest correction of the coronal alignment, where no soft tissue releases were needed. The majority of the cases fell within the target coronal alignment boundaries by only accommodating the individual laxities, suggesting the aim of FA to restore each knee's pre‐pathological alignment.

**Level of Evidence:**

Level IV.

**Abbreviations**
BMI: body mass index
CPAK: Coronal Plane Alignment of the Knee
CT: computed tomography
FA: functional alignment
FJS: Forgotten Joint Score
HKA: hip–knee–angle
IQR: interquartile range
KSS: Knee Society Score
LDFA: lateral distal femoral angle
MA: mechanical alignment
MPTA: medial proximal tibial angle
OKS: Oxford Knee Score
ROM: range of motion
TKA: total knee arthroplasty

## INTRODUCTION

For decades, the attainment of neutral coronal alignment of the lower limb has been extensively established as a critical determinant of successful total knee arthroplasty (TKA) [22]. This alignment is known to have a significant impact on both the longevity of the implant and the overall clinical outcomes [3, 11, 13]. Nevertheless, patient dissatisfaction rates after TKA can be as high as 20%, with approximately 50% of the patients reporting residual symptoms or functional issues [8, 26, 32]. This phenomenon could be attributed to the use of mechanical alignment (MA) as a standardized method, which fails to account for the natural variability in individual coronal alignment. This limitation has driven researchers to develop more precise methods for classifying coronal alignment to better address the diversity of knee phenotypes [19, 30]. It should be noted that among the population, approximately 10%–15% of TKAs are performed in patients with valgus deformity [33, 37].

In an effort to enhance patient function and satisfaction after TKA, alignment philosophies that adapt implant positioning to individual patient anatomy have been proposed [15, 21, 46]. The introduction of robotic technologies has advanced the quantification and control of implant positioning and balancing targets. Some robot‐assisted platforms provide three‐dimensional preoperative planning using cross‐sectional imaging, which facilitates a comprehensive assessment of bony anatomy in all planes to optimize implant positioning and sizing [25, 42]. The enhanced control over these variables afforded by robotic tools has led to the emergence of a new alignment philosophy named functional alignment (FA) [24, 27, 35]. FA represents an individualized methodology that acknowledges the bony constitutional anatomy while also considering the substantial variability in soft tissue laxity observed among patients. Its application for the valgus phenotype has been recently described, while there are not yet studies evaluating its safety and outcomes in this population [40].

The present study aimed to thoroughly evaluate the FA's safety in terms of complications and revisions, as well as to present the short‐term clinical and radiographic outcomes. It is of note that this represents the first study on patients with valgus deformity undergoing image‐based robotic TKA under the principles of FA. The hypothesis was that this alignment philosophy would reveal minimal complication rates and satisfactory clinical outcomes in valgus patients. Another hypothesis was that the soft‐tissue‐driven FA would demonstrate a correction of the coronal alignment according to each case's pre‐arthritic alignment.

## MATERIALS AND METHODS

**Figure 1:**
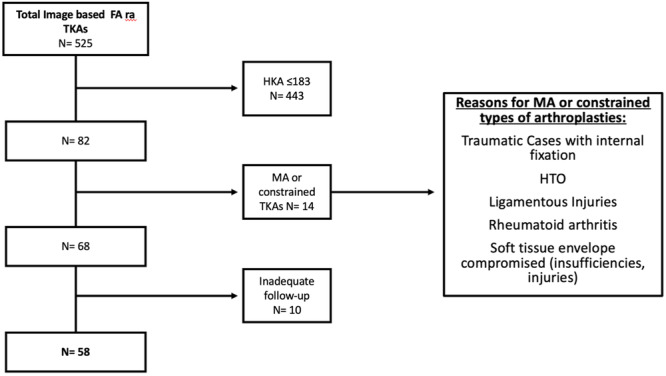
Flow chart of the enrolment process during the study period (April 2021 to October 2023). FA, functional alignment; HTO, high tibial osteotomy; MA, mechanical alignment; TKA, total knee arthroplasty.

The present was a retrospective study of a prospectively maintained database. From April 2021 to October 2023, all patients with valgus coronal alignment (hip–knee–angle [HKA] ≥ 183°) undergoing robotic‐assisted TKA with the FA technique were enroled in this study. The image‐based robotic system Mako 2.0 (Stryker, Mako Surgical Corp.) was used for all procedures. The minimum follow‐up was 1 year, while neutral alignment cases, as well as those in which the MA philosophy was implemented, were excluded. Figure 1 exhibits the enrolment process.

### Clinical evaluation and radiographic analysis

Data collection included demographic features and pre‐ and post‐operative Knee Society Score (KSS) [39]. The Forgotten Joint Score (FJS) [5] and the Oxford Knee Score (OKS) [31] were also assessed at the final follow‐up. The preoperative radiologic analysis included computed tomography (CT) evaluation of the limb and standing full‐length radiographs to assess the hip–knee–angle (HKA), the lateral distal femoral angle (LDFA), and the medial proximal tibial angle (MPTA). According to the measured values, the knees were categorized into nine phenotypes according to the CPAK classification [30].

Post‐operative radiological assessment was performed at 2 and 12 months. Full‐length radiographs were performed at 1‐year follow‐up only and analyzed to assess the HKA, LDFA and MPTA, as well as their classification into CPAK phenotypes. All measurements were performed by two independent investigators (P.G. and C.K.) using tools available in a picture‐archiving communication system and recorded to the nearest 0.1°. Inter‐rater reliability of the measurements was measured using the kappa (*κ*) statistic [23]. The resulting inter‐rater agreement *κ* value was 0.91.

### Surgical technique

The surgical technique followed the principles of the FA philosophy for the valgus phenotype [40]. A medial subvastus approach through a central incision was performed in all cases, without a tourniquet. After accessing the joint, the femoral and tibial pins were positioned as described by Koutserimpas et al. [47]. After extracting the alignment features, the specific laxities of the patient for the medial and lateral compartments were assessed at approximately 10° (to avoid posterior capsule tightness) and 90° degrees flexion for both the medial and lateral compartments.

The goal for a valgus case was to achieve 0 mm gap in extension and up to 1.5 mm and 1 mm gaps in flexion [34] for the lateral and medial compartments, respectively. The thickness of the cuts should never be below 1.5 mm or exceed 10 mm.

Initially, we evaluated the gaps in extension, making corrections by distalizing or proximalizing both the tibia and femur to maintain the joint line height. At this stage, we adjusted the valgus/varus alignment of the implants, adhering to the following boundaries: for the femur, 3° varus to 6° valgus, and for the tibia, 2° valgus to 6° varus. Next, we assessed the gaps in flexion and adjusted the anterior/posterior positioning as well as the internal rotation (IR) and external rotation (ER) of the femur, within a range of 3° IR to 6° ER. The posterior slope of the tibia was set to 3° for the cruciate‐retaining (CR) design and 0° for the posterior‐stabilized (PS) design. Finally, we evaluated anterior notching by increasing the flexion of the femoral implant. We ensured that the combined flexion (femoral flexion plus the tibial posterior slope) did not exceed a total of 10°. Then, a strict evaluation of the matching between the native trochlea and trochlea of the implant as well as the tibia coverage was performed. This technique aimed to avoid any soft tissue release, whenever possible. All patients received either a CR or a PS (in cases of posterior cruciate ligament deficiency or significant flexion contracture), fixed‐bearing implant (Triathlon).

### Ethical approval

All procedures adhered to the ethical standards set by the institutional and/or national research committee, as well as the 1964 Helsinki Declaration and its subsequent amendments or equivalent ethical guidelines. Data collection and analysis complied with the MR004 Reference Methodology established by the Commission Nationale de l'Informatique et des Libertés (Ref. 2229975V0). In line with institutional guidelines, formal patient consent was not required for this type of study.

### Statistical analysis

In this retrospective study with an ‘all‐comers design’, no power analysis was conducted. The Kolmogorov–Smirnov test was used to assess the normality of data distribution. Based on the normality results, either a *t* test or Mann–Whitney *U* test was utilized to compare groups. Complication rates between the groups were compared using the chi‐squared test, with statistical significance defined at *p* < 0.05. Survival analysis of the implants was performed using the Kaplan–Meier method. All analyses were conducted with MedCalc software, version 22.021.

## RESULTS

Sixty‐eight patients met the inclusion criteria. Of these, 58 completed the minimum 1‐year follow‐up and were included for analysis. There were 39 female (67%) and 19 male (33%) patients, with a median age of 70 (interquartile range [IQR] = 64–74.5) years and a mean body mass index (BMI) of 26.6 kg/m^2^ (standard deviation [SD] = 5.4). The median follow‐up was 18 months (IQR = 13.75–27). Demographics of the cohort are reported in Table 1.

**Table 1:** Demographics and phenotypes distribution of the cohort.

M:F (%)	19 (33):39 (67)
BMI (mean, SD)	26.6–5.6
Age (median, IQR)	70, IQR = 64–74.5
CPAK phenotypes (type, no. of knees, %)	I, 0
	II, 1 (2%)
	III, 18 (31%)
	IV, 6 (10%)
	V, 7 (12%)
	VI, 2 (4%)
	VII, 14 (24%)
	VIII, 4 (7%)
	IX, 6 (10%)

Abbreviations: BMI, body mass index; CPAK, Coronal Plane Alignment of the Knee; IQR, interquartile range; SD, standard deviation.

**Figure 2:**
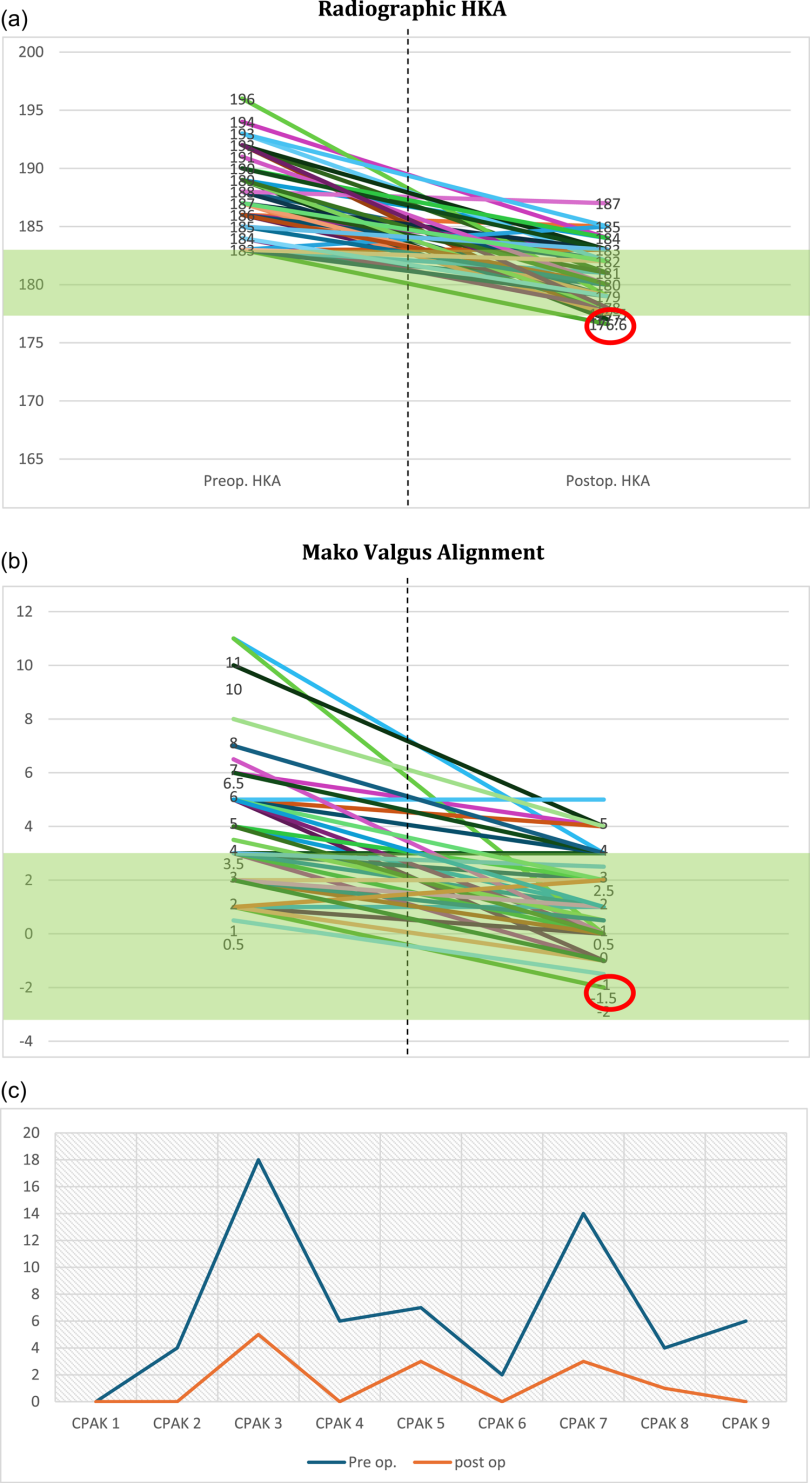
(a) Variation in individual HKA before and after the surgery. The green area represents the goal boundaries of a final coronal alignment for a valgus phenotype. A trend in reducing the coronal deformity is shown. The post‐operative HKA of the aseptic loosening case is highlighted with a red circle. (b) Variation in individual Valgus deformity before and after the surgery according to the Mako System. The final valgus deformity of aseptic loosening case is highlighted with a red circle. (c) CPAK phenotypes variation before and after the surgery. The blue line represents patients categorized by their phenotype preoperatively, while the orange line indicates patients in the same groups who maintained the same phenotype post‐operatively. CPAK, Coronal Plane Alignment of the Knee; HKA, hip–knee–angle.

The final coronal alignment of 86.2% of the patients (50 cases) fell within the goal boundaries (safe zone; HKA = 177–183°). A total of 48.3% of the knees maintained an HKA > 180°, while 79% of the knees moved into a different post‐operative CPAK phenotypes (Figure 2). The femur was more adjusted than the tibia (Table 2). No soft tissue releases were necessary to balance the knees of the present cohort.

**Table 2:** Pre‐ and post‐operative radiological and Mako alignment features.

Studied parameters		Preoperative values	Post‐operative values	*p*
Radiographic evaluation	HKA	187° (IQR = 183.5–189.5)	181.1° (IQR = 179–183)	***p*** < **0.0001**
	LDFA	89° (IQR = 85–94)	90° (IQR = 88–91)	*p* = 0.6145
	MPTA	90° (IQR = 89–92)	90° (IQR = 89–91)	***p*** **=** **0.1579**
Alignment (Mako)	Valgus	3° (IQR = 2–5)	1° (IQR = 0–2)	***p*** < **0.0001**

*Note*: Bold values indicate the statistical significance.

Abbreviations: HKA, hip–knee–angle; IQR, interquartile range; LDFA, lateral distal femoral angle; MPTA, medial proximal tibial angle.

The mean KSS (point 1) improved from 69.5 (IQR = 57–79) to 95 (IQR = 90–97.5) (*p* < 0.001), while the KSS (point 2) improved from 65 (IQR = 50–75) to 94 (IQR = 90–100) (*p* < 0.001). At the final follow‐up, the medina OKS was 45 (IQR = 39–48), while the mean FJS was 93 (IQR = 87.55–100). Pre‐ and post‐operative radiological and clinical outcomes are reported in Table 2 and Table 3, respectively.

**Table 3:** Pre‐ and post‐operative clinical outcomes.

Studied parameters		Preoperative values	Post‐operative values	*p*
ROM	Recurvatum	0° (IQR = 0–0)	0° (IQR = 0–5)	*p* = 0.1435
	Flexion contracture	0° (IQR = 0–0), range = 0–15	0° (IQR = 0–0), range = 0–5	***p*** = **0.0054**
	Flexion	130° (IQR = 120–130)	130° (IQR = 120–130)	*p* = 0.3860
Patient‐reported outcomes	KSS knee	69.5 (IQR = 57–79)	95 (IQR = 90–97.5)	***p*** < **0.0001**
	KSS function	65 (IQR = 50–75)	94 (IQR = 90–100)	***p*** < **0.0001**
	OKS	‐	45 (IQR = 39–48)	‐
	FJS	‐	93 (IQR = 87.55–100)	‐

*Note*: Bold values indicate the statistical significance.

Abbreviations: FJS, Forgotten Joint Score; IQR, interquartile range; KSS, Knee Society Score; OKS, Oxford Knee Score; ROM, range of motion.

**Figure 3:**
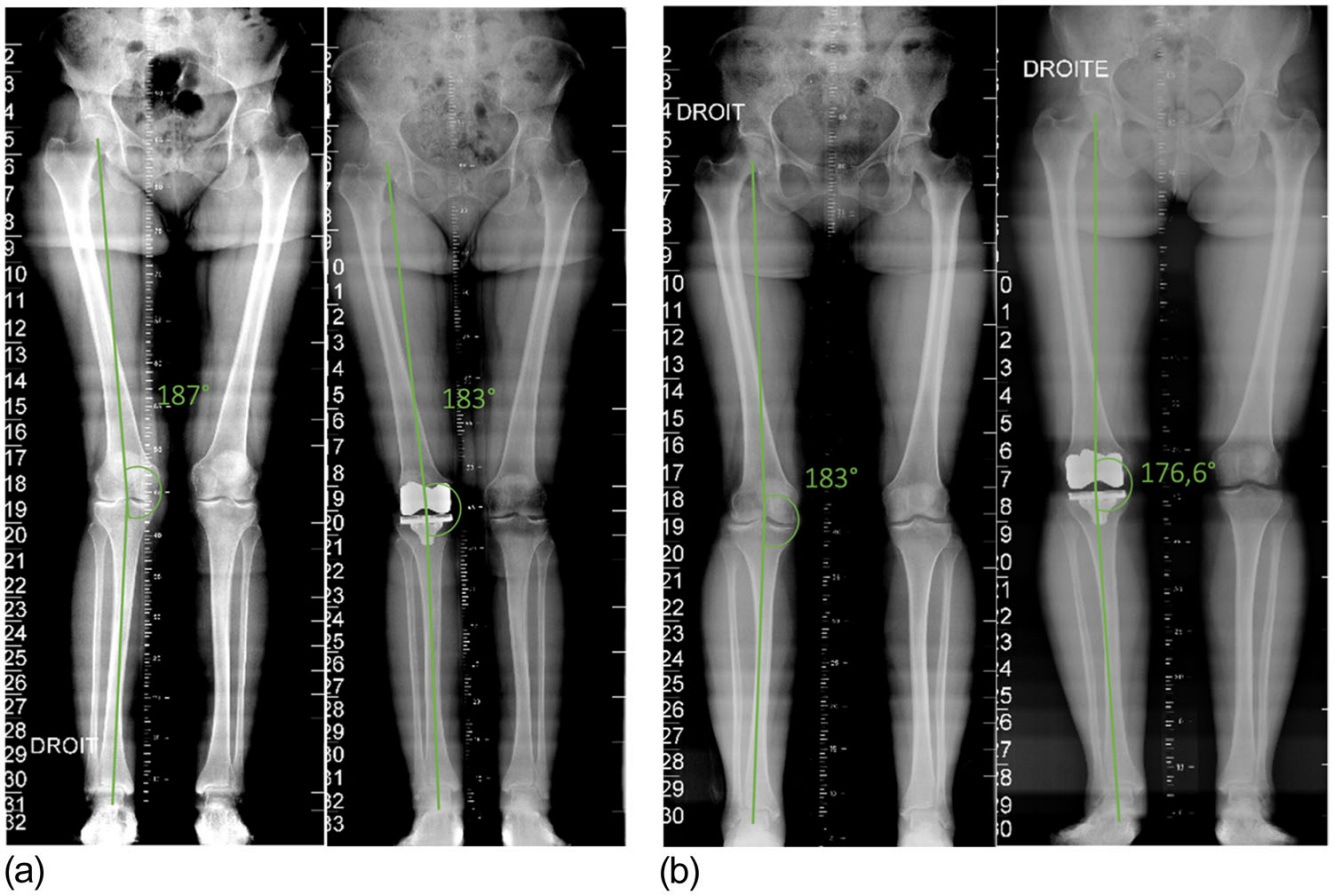
(a) Preoperative and post‐operative coronal alignment of a patient with the final HKA within the expected boundaries. (b) Preoperative and post‐operative coronal alignment of the patient reporting an aseptical loosening of the tibial plateau 7 months after the surgery. A slight out‐of‐the‐target final coronal HKA is shown.

Table 4 exhibits the implants' orientation in three planes in the valgus cases treated according to the functional knee alignment philosophy. Regarding aseptic loosening, one case (1.7%) was recorded concerning the tibial implant (Figures 2 and 3). This failure may be attributed to a significant deviation in the final alignment (183° vs. 176.6°), which also slightly exceeds the target boundaries. However, for this patient, the final alignment from the Mako System reported an HKA of 179° during the tests with the trial components. This discrepancy could be due to a mispositioning after an excessive impact of the definitive implant or due to underestimating the final alignment, since this final evaluation from the robotic system is performed intraoperatively without weight bearing.

**Table 4:** Mako three‐dimensional positioning features of the implants.

Implants positioning	Values
Tibial varus	0.75° (IQR = 0–1.5)
Tibial external rotation	0° (IQR = 0–0)
Tibial slope	0.1° (IQR = 0–1.5)
Femoral valgus	1.5° (IQR = 0.875–3.025)
Femoral external rotation (TEA)	0° (IQR = −0.6 to 1)
Femoral flexion	8° (IQR = 7.4–9.85)

Abbreviations: IQR, interquartile range; TKA, total knee arthroplasty.

**Figure 4:**
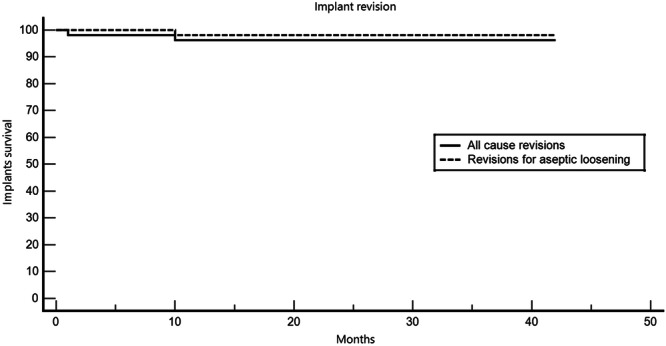
Kaplan–Meyer survival analysis of the cohort.

There was also one early infection that was treated with debridement, polyethylene exchange and antibiotics. Figure 4 shows the Kaplan–Meyer survival analysis of the cohort.

Regarding complications, a case (1.7%) of stiffness that required manipulation under anaesthesia was also recorded. This patient belonged to the final alignment outliers group. There were no cases of patellar instability recorded during the study period.

## DISCUSSION

The main finding of the current study was that FA in patients with valgus deformity undergoing robotic TKA provided satisfactory clinical outcomes in nearly all individuals. Furthermore, most of the cases ended up in the target coronal alignment boundaries (86%), without focusing on the alignment intraoperatively, by just following the individual laxities of the patients.

Post‐operatively, only eight knees exhibited a combined coronal orientation outside the safe goal range of 180 ± 3°. It should be noted that all these knees had a preoperative HKA > 186°. These variations were expected due to the restoration of cartilage and bone loss. The outliers from the safe range (180 ± 3°) likely resulted from the need to accommodate individual laxities and anatomy, which exhibit variability among patients [7, 14, 41]. In these settings, FA seemed to slightly correct just the deformity caused by arthritis, which, if we consider the data coming from the robotic system used, should be around 2°. Therefore, patients with an HKA over 186 could not end up in the safe zone after this procedure, also proving that this technique aimed at restoring the pre‐arthritic knee alignment instead of correcting systematically the deformity into a predefined range of HKA. Interestingly, our results suggest that the mean preoperative HKA was around 7° valgus, but just 3° when using the robotic system. This difference might be due to the reduction in coronal deformity between weight‐bearing (preoperative standing full‐length radiographs) and non‐weight‐bearing (intraoperative CT scan) assessments.

While other personalized techniques are influenced by the specific final coronal alignment intraoperatively for the positioning of the implant [43, 45], the individual changes in alignment angles indicate that the described technique consistently achieved the target boundaries just accommodating the individual laxities and anatomy of the patients. In this perspective, the laxities, when non‐pathological, could be defined as the ‘DNA of the knee’, containing information that could lead to the pre‐pathological and natural alignment of the patient. The present study highlighted a significant variation in the CPAK phenotype before and after surgery (79%). A study by Bertugli et al. demonstrated that this variation did not adversely affect functional outcomes in a cohort of 201 patients treated with image‐based robot‐assisted TKA with FA [2].

The pursuit of improved patient satisfaction has led some authors to suggest methodological adjustments, promoting surgical techniques that better align with pre‐arthritic anatomy of the patients [6, 29, 38].

The FA technique requires the use of a robotic platform capable of intraoperative evaluation of mediolateral laxities in both extension and flexion. The image‐based robotic system offers these functionalities while maintaining minimal system‐related complications and a very low risk of pitfalls [10, 47]. This approach has been associated with improved component placement accuracy and reduced soft tissue damage compared to conventional techniques [1, 28, 44].

Regarding personalized limb alignment final target after surgery, Rak et al. [36] reported significantly lower median FJS and OKS following TKA with MA in patients whose limb phenotype changed by more than one HKA category (3°) [18], compared to those whose limb phenotype remained within a single HKA category of their original alignment. These results support the hypothesis that a tailored alignment positioning of the components might enhance clinical outcomes in TKA.

Selecting a personalized alignment technique for a varus phenotype is widely increasing in popularity due to its not uniform or satisfactory outcomes across all phenotypes [12] However, in cases of valgus deformity, the majority of surgeons prefer performing an MA technique to minimize the risk of complications. Studies on kinematic TKA have reported the necessity of ligament release [9, 20], particularly in cases involving valgus knees, indicating that the need for additional balancing is not entirely resolved in these patients just reestablishing their pre‐arthritic knee morphology. In a similarly sized study, Howell et al. noted that 17% of their patients required the release of lateral structures [20]. Although there were no early failures observed in this cohort, we are unable to draw conclusions regarding the impact of this technique on long‐term function or survivorship. However, the present study exhibited minimal complication rates and low rates of revisions in a median of 1.5‐year follow‐up. Greenberg et al. [16] reported two intraoperative tibial fractures, one partial avulsion of the patellar tendon, two cases of post‐operative peroneal nerve palsy, and two infections in a valgus cohort of patients treated with MA through a lateral approach. The clinical results were satisfactory when compared to the patients treated with the medial parapatellar approach, but still inferior to the ones reported in our cohort (post‐operative KSS point 1 87 vs. 95 and post‐operative KSS point 2 64 vs. 94). However, this study considered a different surgical approach, a significantly higher pre‐operative valgus angle, a higher average age and BMI, and a lower KSS and ROM prior to surgery, in comparison to the present cohort.

Accordingly, Gunst et al. [17] reported post‐operative KSS point 1 of 88.8 and a KSS point 2 of 74.3 in patients with valgus deformity between 3° and 10° treated with MA through a lateral parapatellar approach, with five fractures, three infections, two cases of skin necrosis as early complications. However, they presented a wider and more heterogenous cohort of patients (315 cases). To our knowledge, this represents the first study focusing on FA in patients with valgus deformity reporting its surgical technique and short‐term outcomes.

The present study has some limitations. First, as a single‐centre retrospective study, it does not possess the robustness of a prospective, multicenter investigation. Nevertheless, it facilitates homogeneous data collection and assessment of a population of osteoarthritic patients treated using the same surgical technique by a limited number of experienced surgeons. This design enhances the immediate interpretation of the results, minimizing the influence of external variables. Also, a single implant was used underlining a possible lack of reproducibility of these results for patients with different prosthesis configurations. Future studies could also include data on the potential data from the robotic system's software, such as the pre‐ and post‐operative laxities and the individual femoral and tibial thickness of the bone cuts. This could highlight which adjustments are essential in the setting of FA in valgus patients. Finally, an analysis of variation in a less simplified classification of knee phenotypes [4, 19] should be conducted to gain more personalized insights into the robotic‐assisted FA TKA technique.

## CONCLUSION

FA in patients with valgus deformity undergoing image‐based robotic TKA seems to be a safe procedure, with minimal revision and complication rates at a minimum of 1‐year follow‐up. Furthermore, this technique seems to offer satisfactory outcomes in the short term. This procedure showed a slight correction in coronal alignment after the procedure, while most of the cases ended up in the target coronal alignment boundaries (safe zone), by just accommodating the individual laxities, thus suggesting the aiming for the pre‐pathological individual alignment of every knee, whenever possible. Further studies are needed to draw conclusions regarding the impact of this technique on long‐term clinical outcomes and survivorship.

## AUTHOR CONTRIBUTIONS

Pietro Gregori, Christos Koutserimpas and Sebastien Lustig conceived the idea and were responsible for revising the manuscript. Pietro Gregori was responsible for writing the manuscript and qualified as the corresponding author. Vasileios Giovanoulis was responsible for data acquisition and analysis and realization of figures and tables. Christos Koutserimpas was responsible for statistical analysis. Cécile Batailler and Elvire Servien were responsible for conceptualization and supervised data acquisition and analysis. Cécile Batailler, Elvire Servien and Sebastien Lustig were responsible for reviewing and critically revising the manuscript. All authors have given final approval for the version to be published.

## CONFLICT OF INTEREST STATEMENT

Cécile Batailler: Consultant for Smith and Nephew and Stryker. Elvire Servien: Consultant for Smith and Nephew. Sébastien Lustig: Consultant for Heraeus, Stryker, Depuy Synthes, Smith and Nephew. Institutional research support to Lepine and Amplitude. Editorial Board for Journal of Bone and Joint Surgery (Am). The remaining authors declare no conflicts of interest.

## ETHICS STATEMENT

All procedures adhered to the ethical standards set by the institutional and/or national research committee, as well as the 1964 Helsinki Declaration and its subsequent amendments or equivalent ethical guidelines. Data collection and analysis complied with the MR004 Reference Methodology established by the Commission Nationale de l'Informatique et des Libertés (Ref. 2229975V0). In line with institutional guidelines, formal patient consent was not required for this type of study. All patients provided legitimate, informed consent.

## Data Availability

The data that support the findings of this study are available from the corresponding author (Pietro Gregori) upon reasonable request.
